# Hypoxia-Induced Inflammation in In Vitro Model of Human Blood–Brain Barrier: Modulatory Effects of the Olfactory Ensheathing Cell-Conditioned Medium

**DOI:** 10.1007/s12035-024-04517-6

**Published:** 2024-10-07

**Authors:** Aleksandra Agafonova, Alessia Cosentino, Nicolò Musso, Chiara Prinzi, Cristina Russo, Rosalia Pellitteri, Carmelina Daniela Anfuso, Gabriella Lupo

**Affiliations:** 1 Department of Biomedical and Biotechnological Sciences, School of Medicine, University of Catania, 95123 Catania, Italy; 2 CNR-IRIB: Institute for Biomedical Research and Innovation, National Research Council, 95126 Catania, Italy

**Keywords:** Hypoxia, Inflammation, Blood–brain barrier, Human brain microvascular endothelial cells, Olfactory ensheathing cells

## Abstract

Hypoxia compromises the integrity of the blood–brain barrier (BBB) and increases its permeability, thereby inducing inflammation. Olfactory ensheathing cells (OECs) garnered considerable interest due to their neuroregenerative and anti-inflammatory properties. Here, we aimed to investigate the potential modulatory effects of OEC-conditioned medium (OEC-CM) on the response of human brain microvascular endothelial cells (HBMECs), constituting the BBB, when exposed to hypoxia. HBMECs were utilized to establish the in vitro BBB model. OECs were isolated from mouse olfactory bulbs, and OEC-CM was collected after 48 h of culture. The effect of OEC-CM treatment on the HBMEC viability was evaluated under both normoxic and hypoxic conditions at 6 h, 24 h, and 30 h. Western blot and immunostaining techniques were employed to assess NF-κB/phospho-NF-κB expression. HIF-1α, VEGF-A, and cPLA_2_ mRNA expression levels were quantified using digital PCR. ELISA assays were performed to measure PGE2, VEGF-A, IL-8 secretion, and cPLA_2_ specific activity. The in vitro formation of HBMEC capillary-like structures was examined using a three-dimensional matrix system. OEC-CM attenuated pro-inflammatory responses and mitigated the HIF-1α/VEGFA signaling pathway activation in HBMECs under hypoxic condition. Hypoxia-induced damage of the BBB can be mitigated by novel therapeutic strategies harnessing OEC potential.

## Introduction

Multiple events such as ischemic stroke, hypoxic-ischemic encephalopathy, neurodegenerative diseases, brain tumors, and brain injuries can determine hypoxia, a state of low oxygen levels [1].

Hypoxia exerts significant effects on the blood–brain barrier (BBB), compromising its integrity and increasing permeability. The BBB acts as a biological interface that separates the circulating blood from the central nervous system (CNS), regulating movement of molecules to maintain CNS homeostasis [2]. Structurally, the BBB is formed by brain microvascular endothelial cells interconnected via tight junctions (TJs). Furthermore, interactions with other neurovascular unit components like astrocytes, pericytes, and perivascular microglia contribute to BBB integrity and functionality [3]. Pericytes envelop the abluminal surface of capillaries, providing physical and metabolic support, as well as expressing barrier properties. Astrocytes also play a crucial role in shaping the cellular profile of the BBB, guiding the development of the BBB towards its mature phenotype [4, 5].

The core mechanism through which cells respond to low-oxygen environments involves hypoxia-inducible transcription factors (HIFs), pivotal for sensing hypoxia, inducing metabolic changes, regulating proliferation, and controlling inflammation [1]. Notably, HIF-1α accumulation drives angiogenesis by inducing the transcription of vascular endothelial growth factor (VEGF). This promotes endothelial cell migration towards hypoxic regions, crucial for new blood vessel formation and localized oxygen supply [6]. Compelling evidence has suggested that VEGF is strongly induced following HIF-1α activation after cerebral ischemic injury. VEGF upregulation determines greater capillary permeability, resulting in BBB disruption [7].

Hypoxia signaling cross talks with different cellular pathways and participates in multiple biological processes. Here, we have investigated the role of nuclear factor kappa-B (NF-κB) pathway, a key inflammatory response that promotes HIF1-α transcription [8]. NF-κB is a transcription factor that regulates a diverse group of genes including inflammatory cytokines, immune receptors, and stress response genes [9]. Normally sequestered in the cytoplasm by inhibitory kinases known as IκBs, NF-κB is liberated upon phosphorylation, ubiquitination, and subsequent degradation of IκBs by proteasomes upon exposure to hypoxia [1, 10].

Several studies have reported a crosstalk between NF-κB, HIF-1α, and VEGF [11]. In a particular study, hypoxia was identified as a factor influencing the NF-κB-mediated regulation of HIF-1α and VEGF expression in gastric cancer cells. Notably, experimental evidence demonstrated that inhibiting NF-κB suppressed hypoxia-induced angiogenesis, resulting in reduced levels of HIF-1α and VEGF expression. Therefore, activation of the NF-κB/HIF-1α/VEGF pathway exhibits hypoxia-dependent nature and contributes to the promotion of angiogenesis in gastric cancer [12].

Hypoxic environments can induce inflammation as part of the cellular stress response. Inflammatory cascades in hypoxic conditions may involve the activation of diverse signaling pathways and the secretion of pro-inflammatory mediators. Notably, HIF-1α has been implicated in the regulation of interleukin-8 (IL-8), a pivotal chemokine involved in immune responses and angiogenesis [13]. Under hypoxic conditions, HIF-1α activation promotes the transcriptional upregulation of IL-8, which in turn facilitates the recruitment and activation of immune cells, as well as the promotion of angiogenesis [14, 15].

Furthermore, decreased oxygen concentration activates a series of downstream pathways triggering the activation of calcium-dependent cytosolic phospholipase A_2_ (cPLA_2_). cPLA_2_ plays a crucial role in the inflammatory process by catalyzing the release of arachidonic acid (AA) from membrane phospholipids. AA serves as a precursor for various eicosanoids, including prostaglandin E2 (PGE2), which is a potent mediator of inflammation generated through the cyclooxygenase-2 (COX-2)-catalyzed conversion of AA [16]. Significantly, it has been demonstrated that short-term hypoxic exposure enhances endothelial cell proliferation, migration, and tube formation, upregulating COX-2 and VEGF expression and promoting PGE2 release [17]. Additionally, another study suggested a critical role for COX-2-derived PGE2 in HIF-1α regulation and implicating COX-2 inhibitors as potential agents to prevent hypoxia-induced HIF-mediated gene transcription in cancer cells [18]. These findings highlight the interplay between hypoxia-induced signaling pathways and the inflammatory response mediated by PGE2.

Olfactory ensheathing cells (OECs) represent a particular glial cell population existing in the olfactory system, showing properties with both Schwann cells and astrocytes, belonging to peripheral and CNS, respectively. They are in contact with the small non-myelinated axons of the olfactory receptor neurons, accompanying them from the basal lamina of the epithelium to the olfactory bulb [19, 20]. The OECs are a source of different growth factors, such as GDNF, bFGF, NGF, BDNF, CNTF, and neurotrophins NT4 and NT5 [20]; they also express adhesion molecules [21] and numerous markers [20], including nestin [22], a specific stem cell marker. For these characteristics, OECs have drawn considerable interest because they are able to promote axonal regeneration, functional restoration, and remyelination in the injured sites [23], promoting also vascularization [24, 25]. In addition, transplanted OECs intermingle with astrocytes, compared to Schwann cells, which stimulate astrocytic gliosis in lesioned areas [26]. Therefore, OECs could represent a suitable tool for cellular therapy in different neurological disorders and injured CNS.

During their growth, OECs release a variety of signaling molecules, growth factors, and other bioactive substances into the culture medium. Researchers have been particularly interested in exploiting the therapeutic potential of these secreted factors, as they may play a crucial role in promoting neuronal survival, growth, and regeneration [27]. Studying the medium conditioned by OECs (OEC-CM) offers valuable insights into the complex interplay of molecular signals underlying their neuroregenerative properties.

By unraveling the molecular components within the OEC-CM, scientists aim to discover new avenues for enhancing neural repair and facilitating functional recovery in conditions such as spinal cord injury, neurodegenerative diseases, and peripheral nerve damage [28].

Furthermore, several investigations have suggested potential anti-inflammatory properties of OEC-CM. For example, OEC-CM has demonstrated the ability to protect astrocytes from oxidative damage by promoting cell survival and reducing apoptosis of damaged cells [29].

In our study, we hypothesized that OEC-CM, with its mix of secreted factors, could modulate the response of cells comprising the BBB when exposed to a hypoxic environment. This area of research holds relevance for the development of novel therapeutic strategies against neurodegenerative disorders, stroke, or conditions in which hypoxia is involved.

To investigate this hypothesis, we evaluated the effects of OEC-CM on human brain microvascular endothelial cells (HBMECs) under hypoxic conditions. Our findings revealed that OEC-CM attenuated pro-inflammatory responses and mitigated the activation of HIF-1α/VEGF-A signaling pathways in HBMECs.

## Materials and Methods

### Cell Culture

Immortalized human brain microvascular endothelial cells (HBMECs) were purchased from Innoprot (P10361-IM, Elexalde Derio, Spain). HBMECs were cultured in Dulbecco’s modified Eagle medium (DMEM) with GlutaMAX supplement and 10% fetal bovine serum (FBS, Thermo Fisher Scientific) and 1% penicillin/streptomycin (Sigma-Aldrich, Milan, Italy) in a 37 °C humidified atmosphere containing 21% O_2_, 5% CO_2_, and 74% N_2_. Cells were used for experimental procedures at approximately 70% confluence and ranged from passage P1 to P7 to preserve their phenotype for a limited range of passages. For hypoxia treatment, HBMECs were incubated in a hypoxic incubator filled with 1% O_2_, 5% CO_2_, and 94% N_2._ In all experimental conditions, the FBS concentration was reduced to 5%.

### Preparation of Conditioned Medium

Olfactory ensheathing cells (OECs) were isolated from 2-day-old mouse pup olfactory bulbs, dissected in Leibowitz L-15 cold medium (Sigma-Aldrich, Milan, Italy) and then digested in Minimum Essential Medium-HEPES (MEM-H, Sigma-Aldrich, Milan, Italy), with the addition of 2.5% trypsin (Sigma-Aldrich, Milan, Italy) and 0.1% collagenase (Invitrogen, Milan, Italy). Successively, OECs obtained were seeded in flasks and cultures in DMEM with GlutaMAX supplemented with 10% FBS and 1% penicillin/streptomycin (Sigma). To reduce the number of dividing fibroblasts, cytosine arabinoside (10^−5^ M; Sigma), an antimitotic agent, was added for 24 h. OECs were characterized by immunocytochemistry using S-100/p75: the percentage of S-100/p75 positive cells was about 85–90% (data not shown). OECs were grown at 37 °C in fresh DMEM/FBS, replaced twice a week. When the cells reached the III-IV passage, OEC-CM was collected and filtered through a membrane filter (0.2-µm pore size, hydrophilic nylon membrane, 47-mm diameter; Millipore, cat. GNWP04700) in order to remove debris [30], aliquoted, and stored at − 20 °C until further use.

### Cell Counting Kit 8 Assay

Cell viability was determined by Cell Counting Kit 8 assay (CCK-8) assay (Sigma-Aldrich, St. Louis, MO, USA). Briefly, cells were plated in 96-well plates with a density of 1.5 × 10^4^ cells per well. After 24 h, cells were treated with OEC-CM under both normoxic and hypoxic conditions for 6 h, 24 h, and 30 h. Subsequently, cells were incubated at 37 °C for 3 h with 100 μL DMEM containing 10 μL CCK-8 solution. Absorbance at 450 nm was then measured in a microplate reader (Synergy 2-BioTek). Each assay was carried out in triplicate, from three independent experiments.

### Immunoblot Analyses

For immunoblot analyses, cells were seeded onto 25-cm^2^ flasks at a density of 1.5 × 10^6^ cells per flask. After 24 h, cells were treated with OEC-CM under both normoxic and hypoxic conditions for 6 h and 30 h. Total protein extracts were obtained by lysis in RIPA buffer (20,188, EMD Millipore Corporation, Temecula, CA, USA) containing cocktails of protease inhibitors (Protease Inhibitor Cocktail Set III EDTA—Free, 539,134, 797 EMD Millipore Corporation) and phosphatase (Phosphatase Inhibitor Cocktail 2, P5726, and Phosphatase Inhibitor Cocktail 3, P0044, Sigma-Aldrich, St. Louis, MO, USA). The protein concentration was determined by the BCA assay (BCA Protein Assay Kit; sc-202389, Santa Cruz Biotechnology, Santa Cruz, CA, USA).

Protein extracts (30 μg) were subjected to immunoblot blot analysis using conventional SDS-PAGE gel electrophoresis and protein transfer to nitrocellulose membranes (Packs 0.2 μm Trans-Blot Turbo Mini Nitrocellulose Transfer Tube; 1,704,158, 806 Bio-Rad Laboratories).

Non-specific binding was blocked with TBST containing 5% BSA for 1 h at room temperature before incubating overnight with the following primary antibodies and dilutions: NF-κB (1:1000; ab32536, Abcam Boston, MA, USA), pNF-κB (1:1000; ab278777, Abcam), and β-actin (1:1000; ab8226, Abcam) as loading control. Secondary anti-rabbit antibody was used for the subsequent incubation (1:10,000; ab6721, Abcam) for 1 h at room temperature. Bands were detected by enhanced chemiluminescence (ECL Super-Signal West Dura Extended Duration Substrate; 34,075, Thermo Fisher Scientific) using Chemi-Doc touch imaging system (Bio-Rad, Hercules, CA, USA). Densitometric analyses of the blots were performed using ImageJ software (version 1.52a, National Institutes of Health, Bethesda, MD, USA).

### Immunocytochemistry

For immunocytochemical analyses, HBMECs, after 6 h and 30 h both in normal and hypoxic conditions, were incubated with the following antibodies: pNF-κB and NF-κB. Cells were fixed by 4% PFA in 0.1 M phosphate-buffered saline (PBS) for 30 min. After washing in PBS, the cell membranes were permeabilized with a solution of 5% normal goat serum (NGS) and PBS containing 0.1% Triton X-100 (PBS-Triton) at room temperature for 30 min. The cells were incubated overnight at 4°C with the following primary antibodies: NF-κB (ab32536, Abcam) and pNF-κB (ab278777, Abcam). Secondary antibodies Cy3 anti-rabbit (1:500, Jackson ImmunoResearch©, Cambridge, UK) were used to visualize primary antibodies for 1 h at room temperature and in dark condition. The immunostained coverslips were analyzed with a fluorescence microscope (Carl Zeiss©, Jena, Germany) and images were captured with the AxioVision imaging system. In all cell cultures where primary antibodies were omitted, no specific staining was observed.

### Extraction of Total Mrna and Cdna Synthesis

Total cellular mRNA was extracted using QIAzol reagent (79,306, QIAGEN Inc., Valencia, CA, USA) following the manufacturer’s instructions. Both quantity and quality of mRNA have been assessed through chip-electrophoresis using the Agilent Small RNA Kit (cat. no. 5067–1548, Agilent Technologies) on the Agilent 2100 Bioanalyzer System. The RNA Integrity Number (RIN) was around 10 for all the samples, indicating the intact profile of mRNA. Subsequent reverse-transcription into cDNA was carried out using the QuantiTect Reverse Transcription Kit (205,313, QIAGEN Inc.).

### Digital PCR

Digital PCR (dPCR) was performed using QIAcuity EG PCR Kit (cat. no. 250111, QIAGEN) and 8,5 k-partitions nanoplates (cat. no. 250021, QIAGEN) on QIAcuity One platform. The set of primers is reported in Table 1. Thermal cycling and imaging were performed following the manufacturer’s instructions.

**Table 1:** Set of primers used for dPCR

Gene	Sequence (5′-3′)
HIF-1α	Fw: GTCGGACAGCCTCACCAAACAGAGC
	Rv: GTTAACTTGATCCAAAGCTCTGAG
VEGF-A	Fw: ATCTTCAAGCCATCCTGTGTGC
	Rv: GAGGTTTGATCCGCATAATCTG
cPLA2	Fw: CTCTTGAAGTTTGCTCATGCCCAGAC
	Rv: GCAAACATCAGCTCTGAAACGTCAGG

### PGE2, VEGF, and IL-8 Release in Cell Media

The supernatants from HBMECs treated with OEC-CM were collected after 6 h and 30 h under both normoxic and hypoxic conditions. Aliquots were then utilized for the estimation of PGE2, VEGF, and IL-8 levels using commercially available kits, following the manufacturer’s instructions (PGE2 kit from Cayman Chemicals Co., Ann Arbor, MI, USA; VEGF and IL-8 kits from R&D Systems Inc., Minneapolis, MN, USA). Each sample was analyzed in triplicate.

### cPLA_2_ Activity

For PLA_2_ activity (cPLA_2_ assay kit, Cayman, 765,021 Ann Arbor, MI, USA), equal amounts of cell HBMEC lysates were incubated in a 96-well plate with the substrate arachidonoyl thio-phosphatidylcholine (ATPC), as previously reported [31]. The results were expressed as pmol of ATPC hydrolyzed per minute and per milligram protein (pmol/min/mg). Each sample was analyzed in triplicate.

### Tube Formation Assay

In vitro formation of capillary-like structures was studied in Matrigel Basement Membrane Matrix system (BD Discovery Labware, Bedford, MA, USA), as previously described [32]. Briefly, HBMECs were seeded on Matrigel at a density of 1.5 × 10^4^ cells per well and incubated in a total volume of 100μL of DMEM or OEC-CM under normoxic and hypoxic conditions for 6 h.

Matrigel provided a three-dimensional support that promoted the formation of a tube-like structure network that was suggestive of in vivo capillaries. Cell organization on Matrigel was observed using a phase-contrast microscope. Pictures from randomly selected fields were processed using ImageJ software. Each experimental condition was run in triplicate from three independent experiments. ImageJ’s Angiogenesis Analyzer was used to analyze the length and number of total master segments and branches.

### Statistical Analysis

All results were reported as mean ± S.E.M. or ± S.D. of three independent experiments conducted in triplicate. One-way or two-way ANOVA was used for statistical comparison between means where applicable. Differences between groups were considered significant for *p*-value ≤ 0.05.

## Results

### HBMEC Proliferation and Viability

**Fig. 1:**
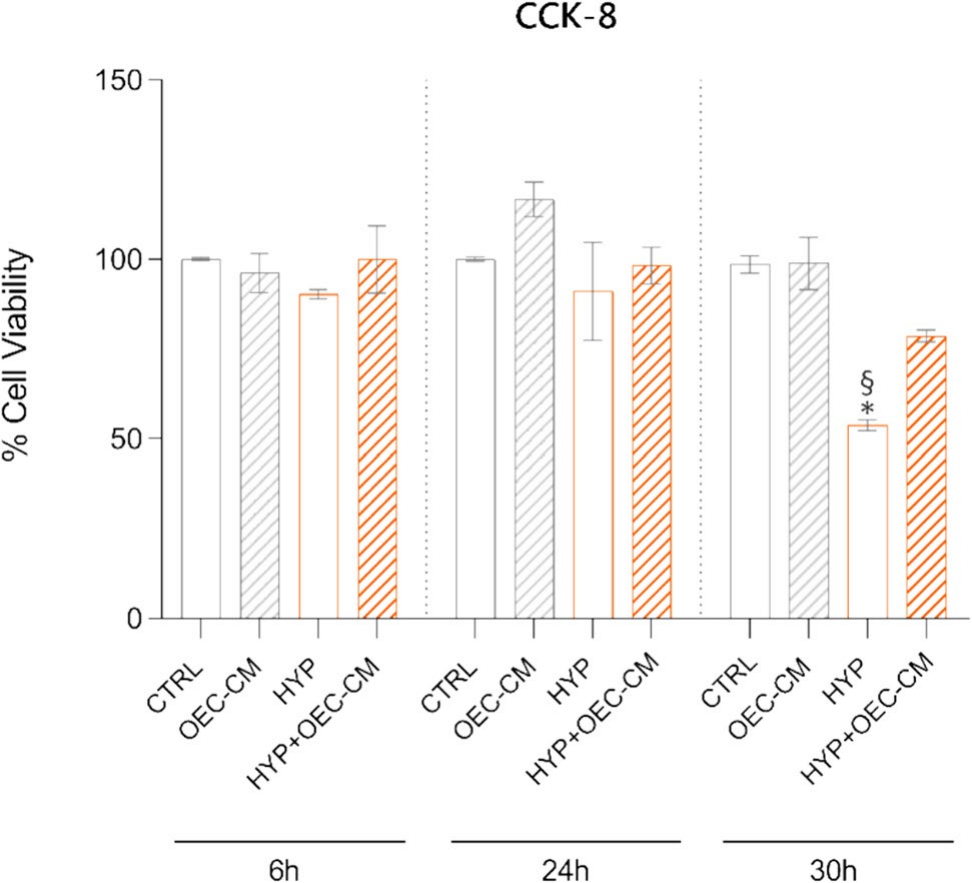
Effect of olfactory ensheathing cell conditioned medium (OEC-CM) on HBMEC viability under normoxic (21% O_2_, 5% CO_2_, and 74% N_2_) or hypoxic conditions (HYP) (1% O_2_, 5% CO_2_, and 94% N_2_). The CCK-8 assay was employed to assess the proliferation and viability of HBMECs at different time points (6 h, 24 h, and 30 h) under normoxic and hypoxic conditions. Bars represent means ± SD of three independent experiments, each performed in triplicate (*n* = 3). Statistically significant differences, determined by one-way ANOVA, are indicated: *p ≤ 0.05 vs CTRL; § *p* ≤ 0.05 vs HYP + OEC-CM

The effect of OEC-CM treatment on the proliferation and viability of HBMECs was assessed using the CCK-8 assay under both normoxic and hypoxic conditions at different time points (6 h, 24 h, and 30 h) (Fig. 1). At the 6 h and 24 h time points, the viability of HBMECs under hypoxic conditions (HYP) in their basal medium closely resembled that of cells in normoxia (CTRL). However, a notable decrease in viability was observed at the 30 h time point. Incubation with OEC-CM under hypoxic conditions (HYP + OEC-CM) at 6 h and 24 h exhibited results comparable to their respective controls in hypoxia, while at 30 h, the viability increased by 1.46-fold compared to the corresponding hypoxic control. Results indicate a significant impact of OEC-CM treatment on HBMEC viability, particularly under long-term hypoxic conditions. Consequently, the 6 h and 30 h time points have been selected for subsequent experiments.

### Modulation of NF-κB/phospho-NF-κB Expression by OEC-CM Under Hypoxic Condition

**Fig. 2:**
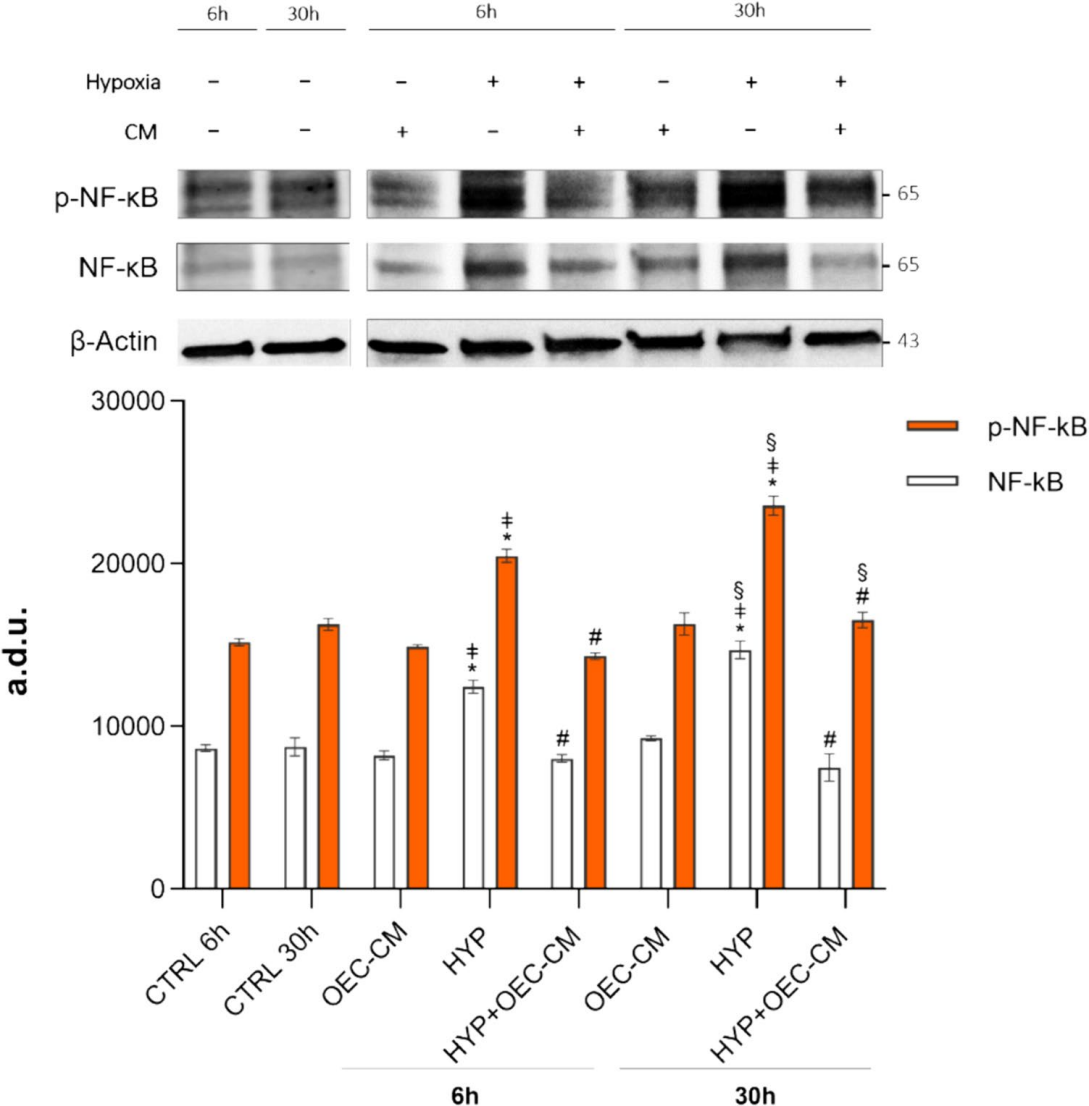
Evaluation of the absolute levels of NF-κB and p-NF-κB expression in HBMECs. Western blot analysis was conducted on samples cultured either in basal culture medium or treated with OEC-CM under normoxic and hypoxic (HYP) conditions for 6 h and 30 h. The analyses were performed on the relevant lysates using specific antibodies against NF-κB and p-NF-κB. Densitometric analysis was carried out using ImageJ software. Bars in the graph represent means ± SD of three independent experiments, each performed in triplicate (*n* = 3). Statistically significant differences, determined by one-way ANOVA followed by Tukey’s multiple comparisons test, are indicated: *p ≤ 0.05 vs respective CTRL at 6 h and 30 h; # *p* ≤ 0.05 vs respective HYP at 6 h and 30 h; ǂ *p* ≤ 0.05 vs respective OEC-CM at 6 h and 30 h; § *p* ≤ 0.05 30 h vs 6 h

To assess the potential effects of OEC-CM treatment under hypoxic conditions in HBMECs, we examined NF-κB/phospho-NF-κB (p-NF-κB) expression. The summarized data of the absolute levels of NF-κB and its phosphorylated form are presented in Fig. 2. There was no significant difference in NF-κB/p-NF-κB expression between basal controls and OEC-CM treatment at 6 h and 30 h time points. Notably, a significant simultaneous increase in NF-κB/p-NF-κB protein expression (by about 1.43- and 1.35-fold, respectively) was observed after 6 h in hypoxia. Comparatively, an even more pronounced elevation of both NF-κB and its phosphorylated form was evident at the 30 h time point (by about 1.68- and 1.45-fold, respectively). Conversely, OEC-CM treatment resulted in a reduction of 35% (NF-κB) and 30% (p-NF-κB) at 6 h, and 50% (NF-κB) and 30% (p-NF-κB) at 30 h in their expression levels.

**Fig. 3:**
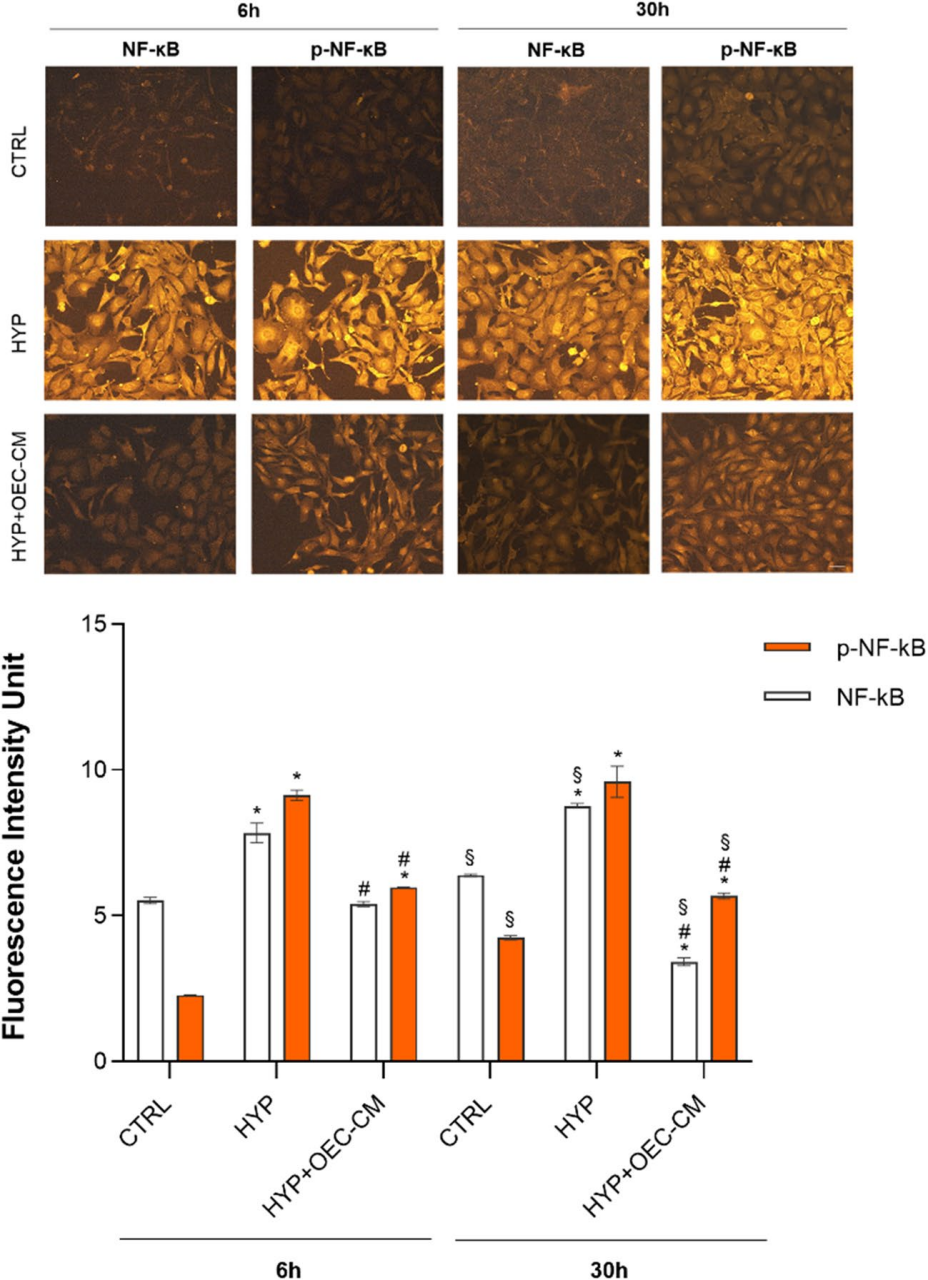
NF-κB and p-NF-κB immunostaining of HBMECs cultured in basal culture medium or treated with OEC-CM under normoxic and hypoxic (HYP) conditions for 6 h and 30 h. Scale bar: 20 µm. Fluorescence intensity quantification was carried out using ImageJ software. Bars in the graph represent means ± SD of three independent experiments, each performed in triplicate (*n* = 3). Statistically significant differences, determined by one-way ANOVA followed by Tukey’s multiple comparisons test are indicated: * *p* ≤ 0.05 vs respective CTRL at 6 h and 30 h; # *p* ≤ 0.05 vs respective HYP at 6 h and 30 h; § *p* ≤ 0.05 30 h vs 6 h

Consistently, we detected a substantial increase in NF-κB/p-NF-κB protein expression after 6 h and 30 h in hypoxia when measured by immunostaining (Fig. 3). OEC-CM treatment mitigated this effect, resulting in a significantly lower protein expression level of both NF-κB and p-NF-κB.

Overall, these results highlight that under hypoxic conditions, a pronounced upregulation of NF-κB expression concomitant with increased phosphorylation occurs. Notably, OEC-CM exhibits a substantial mitigating effect on this heightened expression and phosphorylation, suggesting a modulatory influence of OEC-CM on the NF-κB signaling pathway activation in response to hypoxia.

### HIF-1α, VEGF-A, and cPLA_2_ Gene Expression in HBMECs Treated with OEC-CM Under Hypoxic Condition

**Fig. 4:**
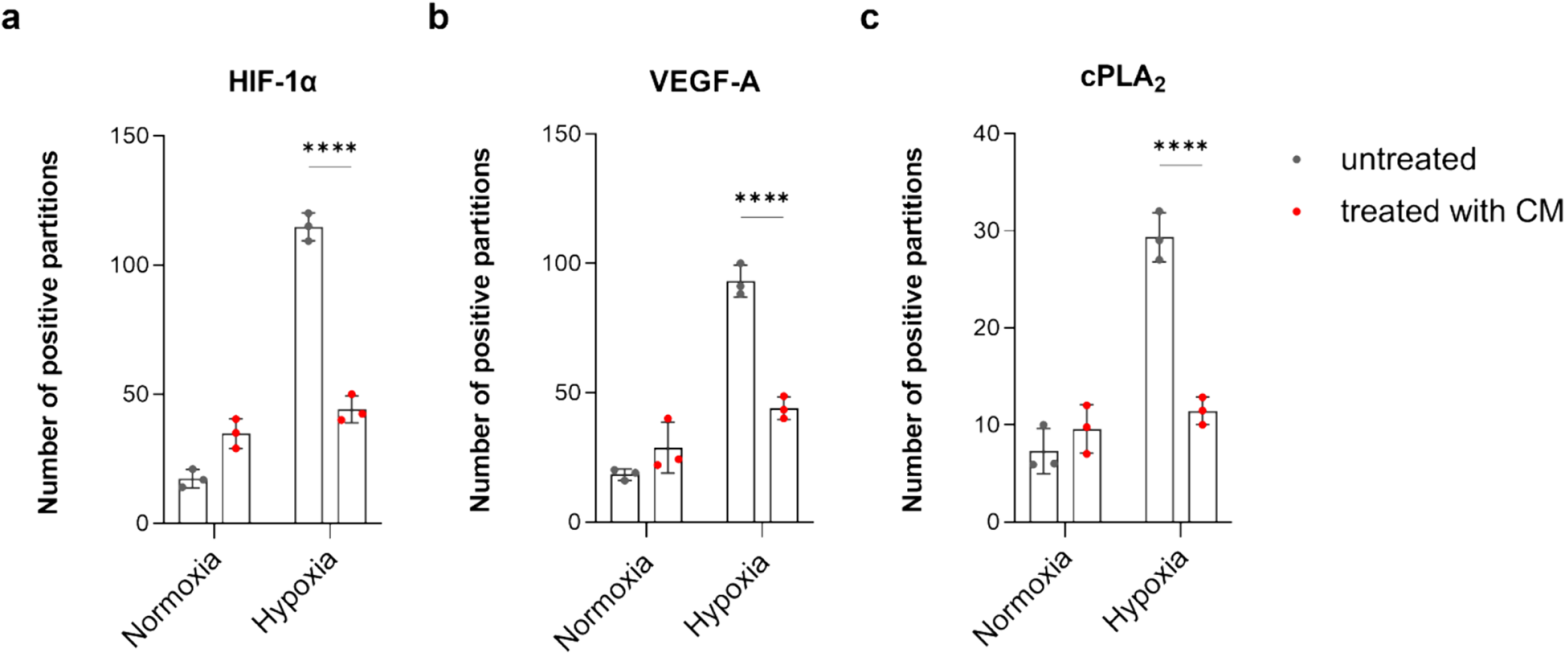
Comparison of **a** HIF-1α, **b** VEGF-A, and **c** cPLA_2_ mRNA levels in the presence of OEC-CM under normoxic or 6-h hypoxic condition in HBMECs. Data were normalized to the GAPDH expression level. All data points are means ± SD (*n* = 3). Statistically significant differences, determined by two-way ANOVA followed by Šídák’s multiple comparisons test, are indicated: ****p < 0.0001

The expression levels of HIF-1α, VEGF-A, and cPLA2 mRNAs were evaluated in HBMECs following treatment with OEC-CM under both normoxic and hypoxic conditions at 6 h (Fig. 4). As expected, HIF expression increased 6.4-fold under hypoxic conditions (Fig. 4a), but the presence of OEC-CM in the oxygen-starved incubation period reduced mRNA levels to control values. The same trend was observed for VEGF-A (Fig. 4b) and cPLA_2_ (Fig. 4c) mRNAs, for which increases of 4.63- and 5.4-fold were observed in hypoxia which, interestingly, were reduced by almost 45.5% and 52%, respectively, in the presence of CM. Notably, the overexpression of these genes was observed in the hypoxic environment, while OEC-CM treatment led to a significant (*p* < 0.0001) decrease in their expression levels.

### OEC-CM Modulates PGE2, VEGF-A, and IL-8 Secretion by Hypoxic HBMECs

**Fig. 5:**
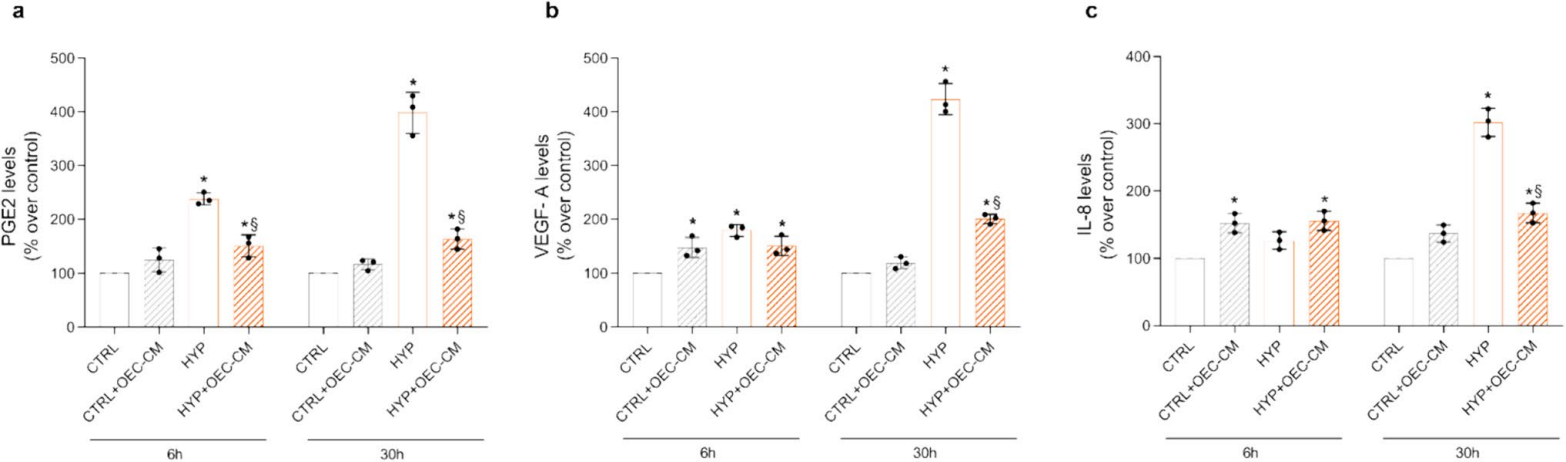
PGE2 (**a**), VEGF-A (**b**) and IL-8 (**c**) levels in media from HBMECs cultured in basal culture medium (CTRL) or treated with OEC-CM under normoxic and hypoxic (HYP) conditions for 6h and 30h. All data points are means ± S.E.M. from three independent experiments (*n* = 3). Statistically significant differences, determined by one-way ANOVA followed by Dunnett’s multiple comparison test, are indicated as follows: * *p* < 0.05 vs respective 6h or 30h CTRL; § *p* < 0.05 vs respective 6h or 30h HYP

Since hypoxia is closely correlated with the inflammatory process in HBMECs [33], we evaluated the levels of PGE2 (Fig. 5a), of the inflammatory cytokine IL-8 (Fig. 5b) and of VEGF-A (Fig. 5c), the growth factor that modulates angiogenesis and aberrant angiogenesis, in hypoxia [34]. Hypoxia induced a significant increase in PGE2 levels at both chosen incubation times. At 6 h, they increased by approximately threefold and at 30 h by 4.2-fold, indicating a strong inflammatory response of HBMECs to the hypoxic insult. Interestingly, the incubation of endothelial cells with OEC-CM reduced the PGE2 secretion by almost 47% at 6 h and by almost 59% at 30 h. The contribution of OEC medium to PGE2 levels was 60.3 ± 7.3 pg/mL. VEGF-A secretion by HBMECs in cell media increased by 1.4-fold at 6 h, and by approximately 3.7-fold at 30 h of hypoxia. The levels of the growth factor did not change at 6 h co-incubation with OEC-CM, but significantly decreased by almost 64% at 30 h of incubation in presence of OEC-CM (the VEGF-A levels found in OEC-CM were 50.7 ± 4.5 pg/mL). IL-8 in cell media showed the same trend of VEGF-A, with a slight but not significant increase at 6 h of hypoxia, and with a significant 3.0-fold increase at 30 h. Once again, OEC-CM co-incubated with HBMECs caused a decrease in IL-8 secretion by 45%. The contribution in IL-8 from OEC-CMs was 54 ± 7.1 pg/mL.

### OEC-CM Modulates cPLA_2_ Specific Activity in Hypoxic HBMECs

**Fig. 6:**
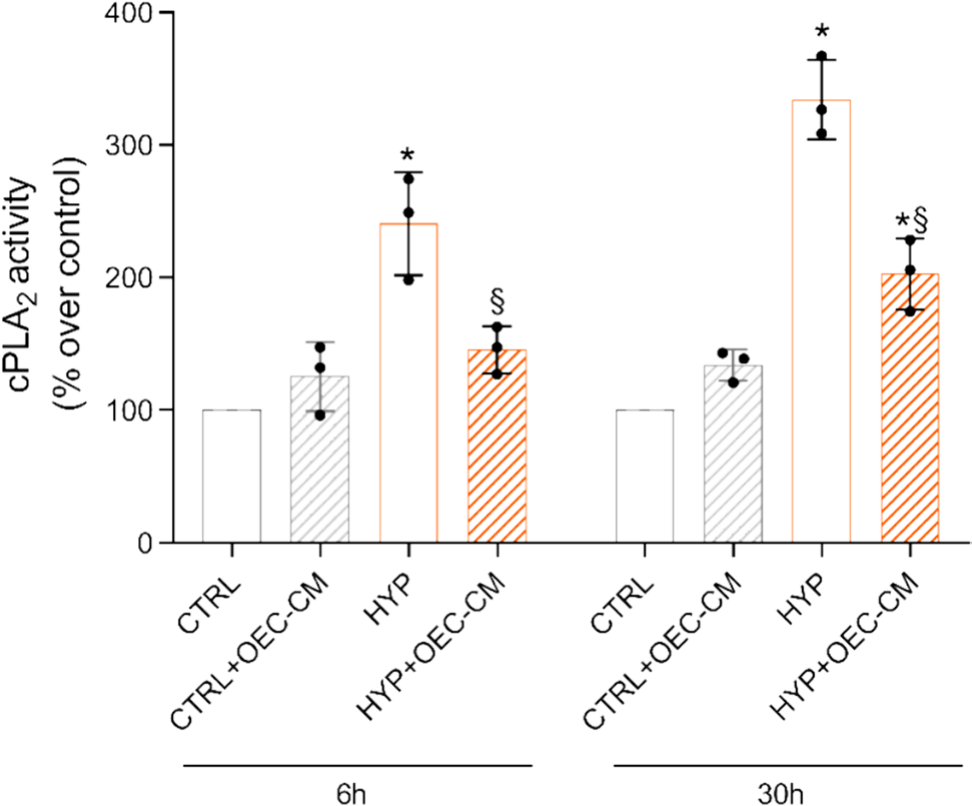
Calcium-dependent cytosolic PLA_2_ specific activity in HBMEC lysates from 6 and 30 h normoxic and hypoxic HBMECs, with or without OEC-CM treatment. All data points are means ± S.E.M. from three independent experiments (*n* = 3). Statistically significant differences, determined by one-way ANOVA, followed by Dunnett’s multiple comparison test, are indicated as follows: * *p* < 0.05 vs respective 6 h or 30 h CTRL; § *p* < 0.05 vs respective 6 h or 30 h HYP

Given that AA is generated from the hydrolytic activity of PLA_2_s on membrane phospholipids and subsequently converted into PGs, such as PGE2 [35], we evaluated the cytosolic calcium-dependent PLA_2_ activity in HBMEC lysates. As shown in Fig. 6, 6-h and 30-h hypoxia-stimulated cPLA_2_ specific activity by 2.35-fold and 3.26-fold, respectively, demonstrating the significant inflammatory insult exerted by the absence of oxygen. OEC-CM caused a decrease in cPLA_2_ activity by 38% and 40% at 6 h and 30 h time points, respectively. The specific activity in control HBMEC lysates was 21 ± 2.3 pmol/min/mg protein. These data showed a sort of modulation of PGE2-inflammation-mediated effect against the hypoxic damage exerted by conditioned medium through the significant reduction of phospholipase activity.

### OEC-CM Counteracts In Vitro Angiogenesis Induced by Hypoxia

While angiogenesis plays a vital role in tissue repair, its dysregulation can contribute to the pathogenesis of various hypoxia-related diseases. Excessive or uncontrolled angiogenesis, triggered by chronic hypoxia, is implicated in the progression of conditions such as cancer, diabetic retinopathy, and inflammatory disorders [36]. For this reason, the tube formation assay was carried out in Matrigel which provide the HBMECs with a three-dimensional scaffold to produce a network of interconnecting tube-like structures in normoxic or hypoxic conditions. Key parameters of the capillary-like patterns were considered to describe the effect of OEC-CM treatment.

**Fig. 7:**
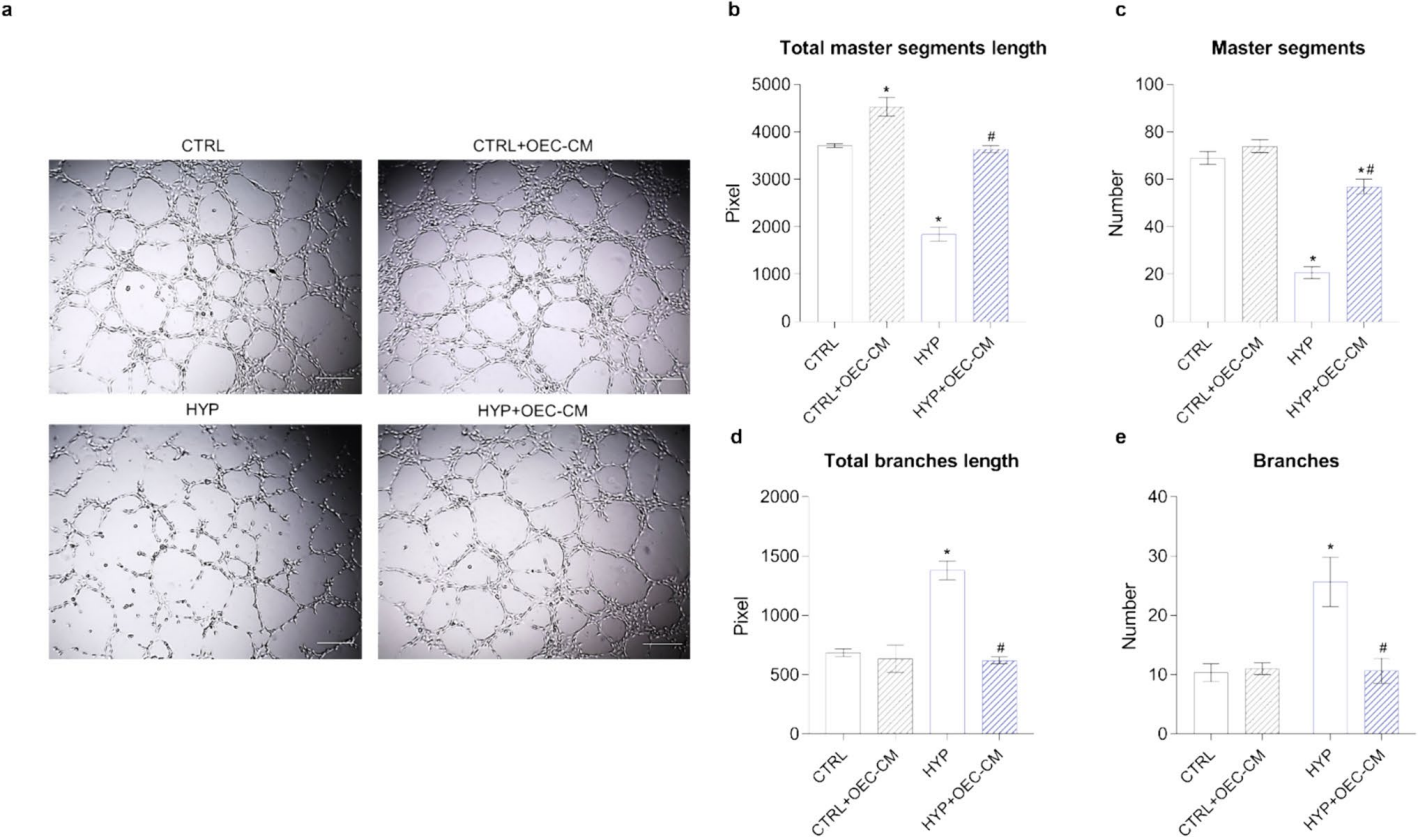
Evaluation of the angiogenic potential of HBMECs in the presence of OEC-CM under normoxic or hypoxic conditions. **a** Representative microphotographs showing three-dimensional cultures in Matrigel of each sample. Magnification: 40 × . Scale bar: 500 μm. The quantification of main parameters describing the capillary network formation after 6 h: **b** total master segments length; **c** number of master segments; **d** total branches length; **e** number of branches. Values are expressed as a mean ± SEM of three independent experiments (*n* = 3). * *p* < 0.05 vs. CTRL; # *p* < 0.05 vs. HYP. One-way ANOVA, followed by Tukey’s multiple comparisons test

As shown in Fig. 7, the following parameters were evaluated to characterize the extension of the capillary network and interconnections inside the network: total master segments length (sum of the length of the detected master segments) (Fig. 7b) and number of master segments (Fig. 7c). Furthermore, total branches length (sum of length of the branches) (Fig. 7d) and number of branches (Fig. 7e), inversely correlating with the strong capillary network formation, were evaluated.

Specifically, quantitative analyses of tube-like structures, as depicted by master segments (Fig. 7b, c), demonstrated a significant reduction in their total length and number by approximately 50% and 71%, respectively, under hypoxic conditions compared to the control (CTRL). Treatment with OEC-CM effectively restored these values associated with tube-like structure formation to levels akin to those observed under normoxic conditions.

The total length and number of branches, inversely correlating with the previous two parameters, confirmed the beneficial effect of OEC-CM treatment. Remarkably, under hypoxic conditions, both the total length and number of branches were significantly increased by 2- and 2.5-fold, respectively. Importantly, treatment with OEC-CM restored these values to levels comparable to the control condition.

In summary, quantitative evaluation of tube formation demonstrated that the capillary network was restored to levels comparable to the control condition following treatment with OEC-CM under hypoxic conditions.

## Discussion

The brain relies on a consistent oxygen supply, facilitated by an intricate network of microvessels that regulate the delivery of oxygen to meet the brain tissue’s dynamic needs. This complex network, known as the blood–brain barrier (BBB), serves as a specialized barrier, both structurally and biochemically, actively regulating the exchange between the bloodstream and the brain.

In adults, an aberrant, not physiological vascular angiogenesis typically occurs as a result of pathological conditions marked by hypoxia or inflammation. The “angiogenic switch” represents one of several tissue responses to hypoxic stress, aimed at enhancing blood circulation and oxygen delivery [37]. The inflammatory cascade triggered by ischemia in nervous tissue includes impairment of energy metabolism, cellular depolarization, excitotoxicity, and severe breakdown of the BBB [38].

The purpose of this study was to observe the effects of OEC-CM in an in vitro model of BBB under hypoxic conditions. We utilized medium derived from OECs, as these cells represent peculiar characteristics. OECs are capable of synthesizing and releasing several growth factors (such as BDNF, NGF, NT3/4, GDNF) and cell adhesion molecules (including laminin, fibronectin, and IL1). These factors are known to be crucial for cell survival and differentiation, exhibiting neuronal protective properties and promoting axon growth [20]. Transplanted OECs have been shown to promote angiogenesis, axonal regeneration, and remyelination in spinal cord injury, fostering neuroplasticity and neuroregenerative effects, as previously reported [39, 40]. Furthermore, our prior research has highlighted the protective effects of OEC-CM on cortical neurons exposed to hypoxia [41]. These properties of OEC-CM prompted us to investigate its protective and anti-inflammatory role on the HBMECs exposed to hypoxic conditions. Since isolation of OECs from patients and their transplantation are not easily feasible, further studies will be needed to investigate the soluble factors released by OECs, which are responsible for the protective effect here demonstrated. Such factors may represent potential therapeutic agents, more readily available, to restore the hypoxia-damaged microvessels. The response of HBMECs to hypoxia, particularly in terms of proliferation and viability, remains contentious, as indicated by several studies [42, 43], and molecular alterations occurring in the endothelium exposed to hypoxia have yet to be fully understood. In our study, HBMECs exhibited resilience when exposed to hypoxia for 6 h and 24 h, but their viability significantly declined after 30 h. Consistent with previous findings from other cell model systems [41], we observed a notable protective effect of OEC-CM on endothelial viability at this latter time point.

Hypoxia and inflammation are coincidental events in tumor growth, ischemia, and chronic inflammation, and the biunivocal correlation between hypoxia and inflammation has been extensively described [44, 45]. These two events are mutually self-amplifying and self-supporting. Inflammation plays a key role in the physiological response to hypoxic stress [1]*,* and hypoxia triggers the expression of numerous inflammatory mediators that initiate survival responses and signal cell damage [46]. If, on the one hand, hypoxia-induced inflammation can play a protective role by triggering the immune response promoting tissue healing, it can contribute to the onset of numerous pathologies if the hypoxic stimulus persists chronically [47].

NF-κB represents a family of regulated transcription factors that are activated simultaneously, leading to gene transcription [48]. The intermediate factors and proteins involved in the NF-κB activation pathway are tumor necrosis factor receptor–associated death domain type 1 (TRADD), tumor necrosis factor receptor–associated factor 2 (TRAF2), the NF-κB-inducing kinase (NIK), mitogen-activated protein kinase/ERK kinase-1 (MEKK), and IκB kinase (IKK) [49]. The NF-κB evoked signaling pathway plays a fundamental role in regulating the expression of genes involved in the control of cell growth, immunity, and inflammation [50]. NF-κB activation is a complex process, and this has led to the description of canonical and non-canonical activation pathways [51]. The activation of the canonical NF-κB pathway occurs through the receptors TNF-α, IL-1, LPS, TLR, and PGE2-EP2, which induce the activation of the IKK complex, composed of two catalytic subunits, IKKα and IKKβ, and a regulatory subunit IKKγ. Once activated, IKKβ phosphorylates IkBα and begins its subsequent degradation, leaving the p50/p65 dimer free for entry into the nucleus and binding to its target genes [52]. The p65 subunit interacts with protein kinase C-δ (PKCδ) in vascular smooth muscle cells, regulating the expression of pro-inflammatory chemokines, thus playing a crucial role in inflammation [53]. Furthermore, it binds to the *Ccl2* and *Ptgs2* genes encoding MCP-1 and COX-2 [54]. The non-canonical NF-κB pathway is mediated by NF-κB-induced kinase (NIK) and is activated when ligands bind to TNFR, CD40, BAFF, and TLβR receptors [55]. NIK phosphorylates IKKα and regulates anti-apoptotic *bcl-2* and *bcl-xl* genes, promoting cell survival [56].

NF-κB is induced by hypoxia, and its activation following hypoxia resulted in a decreased apoptosis and an increased angiogenic sprouting that characterizes tumor growth and spread [57, 58].

In immunoblotting and immunofluorescence experiments, we observed a significant increase in total NF-κB and its phosphorylated/activated form at 6 h and, even more prominently, at 30 h of hypoxia. Treatment with OEC-CM at low oxygen levels resulted in a significant reduction in the expression and activation of the protein, demonstrating a modulation of the NF-κB-mediated inflammatory response in HBMECs exerted by soluble factors released in OEC 48-h secretome at both incubation time points. These findings are consistent with previous studies, which have emphasized the activation of the NF-κB pathway in cells comprising the BBB, such as astrocytes and endothelial cells, in response to inflammatory stimuli [59]. Furthermore, the strong activation of NF-κB has been shown to disrupt tight junctions, resulting in increased permeability of the endothelial cell layer [51, 60].

HIF orchestrates angiogenesis as a response to low oxygen levels, aiming to enhance oxygen supply to tissues through the transcriptional regulation of genes such as VEGF and erythropoietin (EPO) [61]. HIF functions as a heterodimeric protein composed of HIF-1β (whose activity remains constant regardless of oxygen levels) and HIF-1α (the active subunit), which is tightly regulated by oxygen availability [62]. HIF-1α is ubiquitously expressed in nucleated cells, facilitating a rapid response to hypoxia by activating a multitude of genes (more than 150) that modulate various cellular processes, including energy metabolism [63], cell proliferation [64], vascular remodeling, autophagy enhancement, and neo-angiogenesis [65]. In human endothelial cells, the acute onset of hypoxia is characterized by the accumulation of HIF-1α, which promotes initial adaptation, while HIF-2α and HIF-3α levels increase in response to a chronic hypoxic state [66, 67]. Activation of the NF-κB pathway in endothelial cells has been shown to upregulate HIF-1α via a positive feedback loop. HIF-1α activity, in turn, leads to endothelial cell proliferation, angiogenesis, dysfunction, and inflammation [68].

In our experiments, we observed a significant increase in HIF-1α mRNA levels in HBMECs following a 6-h hypoxic incubation. Interestingly, the presence of OEC-CM during the hypoxic incubation period restored HIF-1α expression levels to those observed under normoxic conditions. These results align with previous findings indicating a protective effect exerted by OECs on the survival of neurons exposed to hypoxia in vitro [41].

It has been well established that VEGF expression could be induced by hypoxia, resulting in tumor necrosis and stimulated angiogenesis [69]. In tumor progression, HIF-1α upregulates the expression of related genes encoding all VEGF isoforms, as well as other growth factors (PlGF, FGF, PDGF, and Ang-1), promoting tumor angiogenesis and inducing resistance to drugs [70, 71]. In vitro and in vivo data have shown that low oxygen levels associated with tumor necrosis induced the expression of VEGF, which in turn stimulated vascular endothelial cell proliferation in a paracrine manner, leading to the sprouting of new capillaries [72].

Here, a significant increase of VEGF-A expression was observed in 6-h hypoxic HBMECs, indicating the activation of the HIF-1α/VEGF-A signaling. The presence of OEC-CM during hypoxic exposure led to a significant reduction in VEGF-A expression levels, suggesting a modulation of the HBMEC response by OEC-CM.

Prostaglandins (PGs) serve as mediators of inflammation, influencing blood flow and exerting pro-angiogenic effects [73]. AA, their precursor, is hydrolyzed from membrane phospholipids by various isoforms of phospholipases (PLA_2_s) and subsequently converted into PGs or leukotrienes by cyclooxygenases (COX) and 5-lipoxygenases, respectively. Cytosolic Ca^2+^-dependent PLA_2_ (cPLA_2_), Ca^2+^-independent PLA_2_ (iPLA_2_), and Ca^2+^-dependent secretory PLA_2_ (sPLA_2_) differ from each other for substrate specificity, calcium requirement, translocation to cell membranes and AA release.

It has been shown that cPLA_2_ activation is required for hypoxia-induced VEGF-dependent retinal neovascularization [74, 75]. Previous evidence suggested that the inducible COX-2 modulated angiogenesis by interacting with the VEGF pathway [76].

Similar to the trend observed for HIF-1α and VEGF-A expression levels, 6-h exposure of HBMECs to hypoxia resulted in a significant increase in cPLA_2_ mRNA levels, which were significantly reduced in the presence of OEC-CM. These data were further confirmed by the specific enzyme activity values obtained from ELISA assays. In fact, low oxygen levels led to increased cPLA_2_ enzyme activity in HBMECs at both 6 h and 30 h. Furthermore, treatment with OEC-CM mitigated the AA hydrolyzing activities at both incubation time points considered.

Hence, the data concerning the expression levels of HIF-1α, VEGF-A, and cPLA2 (at both mRNA and specific activity levels) exhibited a consistent trend, collectively indicating a significant inflammatory-proangiogenic response of HBMECs to low oxygen exposure. Concurrently, notable modulatory effects exerted by OEC-CM were observed.

Our previous experiments in human microvascular retinal endothelial cells, demonstrated the strong correlation between NF-κB activation and the ERK/cPLA_2_/COX-2/PGE2 pathway [77]. Moreover, selective inhibition of cPLA_2_ resulted in reduced levels of VEGF and inflammatory cytokines, indicating a synergistic effect between COX upregulation and the synthesis of PGs and VEGF. In that context, PLA_2_ activity appeared to exert its influence both upstream and downstream of VEGF receptor activation, significantly impacting the angiogenic process [78]. Furthermore, in high glucose-stimulated human retinal pericytes, a kind of feed-forward loop between cPLA2/COX-2/PG axis and VEGF appeared to operate [79].

ELISA assays conducted on HBMEC cell media for PGE2 and VEGF-A confirmed the trend observed in mRNA expression levels. At 6 h, and even more prominently at 30 h of hypoxia, significant increases in PGE2 and VEGF-A levels were observed, which were significantly reduced in the presence of OEC-CM.

Hypoxia-induced NF-κB has been shown to upregulate the expression of IL-8 [15, 57], an important chemokine implicated in the induction of angiogenesis [13], thereby contributing to the overall generation of neovascularization in low oxygen conditions. Numerous in vitro and in vivo studies have consistently shown that IL-8 directly modulates endothelial cell proliferation and migration, thus regulating the formation of new blood vessels, both independently and in a paracrine manner [80–82]. Notably, endothelial cells themselves produce IL-8, whose secretion is significantly enhanced during inflammation, infection, and interaction with growing tumors [83, 84]. Specifically, under hypoxic conditions, the activation of HIF-1α promotes the transcriptional upregulation of IL-8, facilitating immune cell recruitment and activation, as well as promoting angiogenesis [14].

In our model system, IL-8 levels increased in the media from HBMECs after 30 h of hypoxia. IL-8 secretion exhibited a decreasing trend, approaching control values when the hypoxic stimulus was applied in the presence of OEC-CM. This underscores the modulatory effect of OECs on the expression and secretion of this chemokine by HBMECs.

The use of the hypoxia model is a well-established tool to stimulate angiogenesis in vitro, exploiting the innate biological mechanism that promotes the formation of new vessels, both in physiological states (e.g., in embryogenesis) and pathological states (e.g., in wound healing, ischemia, and in tumor formation and growth) [85]. In vitro studies performed on cultured brain endothelial cells indicated that hypoxia is a cause of TJ rupture and opening, with the consequent weakening of the BBB [86, 87]. Here, the organization of microcapillaries by HBMECs on Matrigel matrix was significantly affected by hypoxia, with pronounced (*i*) reductions of total tube length and number and (*ii*) increases of total length and number of branches (inversely correlating with the previous two parameters), confirming that hypoxia causes the formation of incomplete and permeable vessels, which inefficiently supply oxygen and nutrients to cells and tissues [88]. In accordance with recent findings from in vivo and in vitro studies, highlighting the pro-angiogenic effects of OECs and their significant potential in promoting angiogenesis when transplanted into injured spinal cord [89], treatment with OEC-CM under hypoxic conditions effectively restored the aforementioned values to those observed under normoxic conditions. This suggests the normalization of the microvascular phenotype by HBMECs under hypoxic conditions in the presence of OEC-CM.

We are aware that our model system has the “non-syngeneicity” limitation. In the hypothesis of moving on to future clinical application, it is crucial to recognize the challenges associated with obtaining olfactory bulbs from patients, which typically involves a transdural or intracranial endoscopic approach, with a high risk of consequent unilateral anosmia. To avoid the challenges of autotransplantation and to ensure an adequate cell number, one potential strategy could involve establishing a bank of cell lines or utilizing allogeneic cells [90].

Furthermore, numerous studies with immuno-incompatible grafts have demonstrated that xenografted OECs immunoprotected with cyclosporine induce axon regeneration and remyelination in the spinal cord [91–94]. Certainly, additional allogeneic sources of OECs may be necessary until research in this field will validate suitable differentiation protocols for generating OECs from patients.

We believe that the various neurotrophic factors responsible for the regenerative capabilities of OECs may also contribute to their modulatory and protective effects against hypoxic damage in endothelial cells. These findings, derived from our in vitro model of the human BBB, align with experiments conducted in vivo, where OEC implantation in a rat spinal cord injury model demonstrated significant efficacy in promoting the formation of new blood vessels alongside axonal regeneration and remyelination, ultimately restoring lost functions in spinal cord lesions [95].

## Conclusions

In conclusion, our study highlights the considerable potential of OEC-CM in mitigating hypoxia-induced BBB in vitro damage.

Our findings reveal a significant anti-inflammatory effect exerted by OEC-CM on HBMECs subjected to hypoxic stress for 6 h and 30 h, mediated by the NF-κB activation/phosphorylation, by the significant increase of cPLA_2_ mRNA levels and enzyme activity, and by PGE2 and IL-8 increased levels in cell supernatants. Furthermore, treatment with OEC-CM during hypoxic conditions significantly mitigated aberrant angiogenesis, as demonstrated by tubulogenic experiments on Matrigel. These effects were corroborated by reductions in HIF-1α and VEGF-A expression levels. Overall, our study underscores the therapeutic potential of OECs in addressing neurodegenerative disorders, stroke, and hypoxia-related conditions.

In this phase of the study, our deliberate focus was solely on examining the impact of hypoxia. Subsequent investigations will encompass the evaluation of both hypoxic exposure and subsequent reoxygenation.

We believe that future studies aimed at characterizing the bioactivity of factors secreted by OECs, which possess anti-inflammatory properties and facilitate physiological angiogenesis in the hypoxic brain, hold significant promise for repairing the functionality of regions affected by inadequate oxygen supply.

## Data Availability

No datasets were generated or analysed during the current study.
